# Impact of the COVID-19 Pandemic on Out-of-Hospital Cardiac Arrests Occurring in the Workplace

**DOI:** 10.7759/cureus.80168

**Published:** 2025-03-06

**Authors:** Yoshio Tanaka, Koichi Tanaka, Tomoyuki Ushimoto, Hideo Inaba

**Affiliations:** 1 Department of Emergency and Disaster Medicine, Graduate School of Medical Sciences, Kanazawa University, Kanazawa, JPN; 2 Department of Emergency Medicine, Kanazawa Medical University, Uchinada, JPN; 3 Department of Emergency Medical Science, Niigata University of Health and Welfare, Niigata, JPN

**Keywords:** cardiopulmonary resuscitation, covid-19, outcome, out-of-hospital cardiac arrest, workplace

## Abstract

Background

The impact of the coronavirus disease 2019 (COVID-19) pandemic on out-of-hospital cardiac arrest (OHCA) occurring in this setting remains unclear.

Objective

The objective of this study is to elucidate the impact of the COVID-19 pandemic on the prehospital characteristics and outcomes of OHCA occurring in the workplace.

Methods

This nationwide observational study in Japan was a retrospective analysis and included 16,364 non-emergency medical service witnessed and adult workplace OHCAs. The characteristics and outcomes of workplace OHCAs were compared between the pre-pandemic period (2016-2019) and the pandemic period (2020-2021). Furthermore, subgroup analyses were performed for workplace location (office vs. non-office) and infection burden region.

Results

During the pandemic period, no significant changes were observed in incidence, public access defibrillation (PAD) provision rates, one-month survival rates, or neurologically favorable survival rates. However, increases were observed for bystander cardiopulmonary resuscitation (CPR) (crude odds rate (cOR), 95% confidence interval (CI): 1.10, 1.02-1.16; P<0.001), particularly compression-only CPR. The multivariable analysis revealed that the impact of the pandemic was similarly seen in an increase in bystander CPR (adjusted OR, 95% CI: 1.14, 1.06-1.22; P<0.001). Furthermore, the monthly changes in only PAD were altered biennially (PAD: P=0.02, bystander CPR: P=0.52, one-month survival: P=0.26, and neurologically favorable one-month survival: P=0.48). Analysis restricted to high-infection burden regions revealed that only the PAD rate decreased (P=0.03).

Conclusion

The COVID-19 pandemic had no impact on OHCA survival in workplaces and had a limited positive impact on bystander responses. This may be attributed to previous positive CPR training experiences and routine preparation for health crises.

## Introduction

In research on the impact of the novel coronavirus disease 2019 (COVID-19) pandemic on the clinical features and outcomes of out-of-hospital cardiac arrests (OHCAs), attention was initially focused on direct effects such as acute respiratory failure and thrombosis [1]. However, with increasing social disruption, the indirect effects became increasingly apparent [2]. Decreased bystander cardiopulmonary resuscitation (CPR) efforts and consolidation and depletion of medical resources [3,4] negatively intervene and disrupt the various stages of the time-dependent chain of survival concept of OHCA lifesaving efforts [5]. Furthermore, it affects OHCA outcomes by causing changes in organizational behavior [6], such as social distancing measures and workplace restrictions. As a result, although the survival rate of patients with OHCA has been increasing worldwide over the past decades [7,8], this pandemic has led to a series of reports of decreased survival rates for the first time [3,9]. In addition, differences in the circumstances in which OHCA is witnessed and where it occurs, such as increased home cardiac arrests, likely contribute to this concerning trend.

Workplaces, where a significant portion of the working population spends substantial time, represent a unique setting vulnerable to the pandemic’s indirect effects. In essential workplaces, the number of people on leave due to infections or close contact quarantines increased dramatically, and in non-essential workplaces, social restrictions, such as limiting the number of people going to the workplace and telecommuting, were implemented. While pre-pandemic studies reported relatively favorable outcomes for workplace OHCAs [10-12], the specific impact of the pandemic on this population remains unknown. Understanding the characteristics and outcomes of workplace OHCAs during this unprecedented pandemic holds both social and economic importance. Furthermore, it may lead to acquiring effective prevention strategies to mitigate the impact of crises such as future infectious disease outbreaks.

Notably, Japan’s relatively low direct COVID-19 mortality rate [13] (146 deaths per million inhabitants in 2020-2021) compared with other developed countries (UK: 2,569; USA: 2,561 per million) makes it an ideal setting to assess the indirect effects of the pandemic specifically. Therefore, this study comprehensively analyzed the national OHCA database in Japan to investigate how the COVID-19 pandemic affected the characteristics and outcomes of workplace OHCAs, with a specific focus on comparing pre-pandemic and pandemic periods.

## Materials and methods

Ethics approval

This study was approved by the Ethical Review Committee of Kanazawa Medical University (No. I-729) and adhered to the Strengthening the Reporting of Observational Studies in Epidemiology (STROBE) guidelines. Consent was obtained to analyze the data that were prospectively collected from the Fire and Disaster Management Agency (FDMA) of the Ministry of Internal Affairs and Communications of Japan. This study uses fully anonymized, routinely collected patient data. Retrospective consent for using these data for research was not feasible or deemed necessary following ethical review.

Study design and patient setting

This study was a retrospective observational analysis spanning January 1, 2016, to December 31, 2021, using a population-based OHCA registry (All-Japan Utstein Registry) based on the standardized Utstein style. Excluding cases of obvious postmortem changes, most patients with OHCA who were treated by emergency medical service (EMS) personnel were transported to a hospital and included in this registry, because EMS providers in Japan are not permitted to terminate resuscitation in the field. Since this Utstein Registry did not contain detailed occurrence location information, we matched it to another nationwide registry encompassing all EMS-transported patients (All-Japan EMS Transport Registry) with the required information. We defined data-matching success for this matching as cases for which three or more of the four time records (occurrence, emergency call, EMS patient arrival, and EMS hospital arrival) were matched, in addition to the prefecture of occurrence. To ensure the accuracy of this matching, patient sex- and age-matches were also checked, retrospectively. The authors wrote the manuscript and vouch for the completeness and accuracy of the data and analyses. Data from these two registries were prospectively collected by EMS personnel. Furthermore, these data were checked for consistency by the computer system belonging to and maintained by the FDMA of the Ministry of Internal Affairs and Communications of Japan.

EMS-witnessed cases and pediatric cases were excluded because the main focus of this study was bystander resuscitation efforts and the working population. The final analysis cohort included adult (≥16 years) OHCAs occurring in workplaces with non-EMS-witnessed (i.e., bystander-witnessed or unwitnessed) OHCA in whom citizens or EMS attempted resuscitation. In this study, we defined workplace OHCA as all OHCAs occurring in the workplace.

Study setting

Japan has a geographic area of approximately 378,000 km^2^ and a population of approximately 126 million people residing there in 2019. All ambulances are public ambulances managed by the FDMA, and patient transport is free of charge. Emergency medical technicians (EMTs) are authorized to use a supraglottic airway device (SGA) and administrate peripheral venous infusion of Ringer’s lactate solution for OHCA cases under the specific advice of a physician over the phone. Only specially trained EMTs are permitted to insert tracheal intubation (TI) and administer intravenous epinephrine [14]. During the pandemic, EMS providers adopted additional standard precautions for OHCA calls, including N95 respirators and isolation gowns.

**Figure 1:**
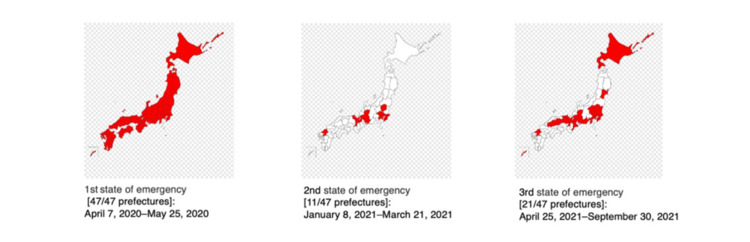
Regions with a high burden of COVID-19 infection in Japan The prefectures in which a state of emergency was declared are indicated in red. Image created by the authors.

On April 7, 2020, the Japanese government declared the first nationwide state of emergency to curb the pandemic’s spread. This included refraining from leaving the house and restricting inter-prefectural travel, except for essential activities. The emergency was declared based on three criteria: (1) rising infection rates; (2) inadequate medical care delivery systems, including the availability of hospital beds; and (3) inadequate surveillance systems that would allow the necessary polymerase chain reaction (PCR) tests to be performed without delay. Emergency declarations were issued for prefectures that met these conditions. During the study period, three emergency declarations occurred: First declaration (47/47 prefectures): April 7, 2020-May 25, 2020; second declaration (11/47 prefectures): January 8, 2021-March 21, 2021; third declaration (21/47 prefectures): April 25, 2021-September 30, 2021 (Figure 1).

In this study, we conducted subgroup analyses by defining prefectures under these state-of-emergency declarations as “high-burden regions” for COVID-19 infection and others without state-of-emergency declaration due to relatively mild infections as “low-burden regions”.

The workplaces were categorized as follows: offices, indoor construction sites, outdoor construction sites, factories, agricultural areas, and others. Office workers were less likely than other workers to be essential workers, and flexible work arrangements, such as remote work, were common during the pandemic. Therefore, offices may be more susceptible to the pandemic, and for subgroup analysis, workplaces were further categorized as “office” and “non-office”. Offices included business, industry, and factory offices. Non-office included agricultural land, aircraft maintenance, factory warehouses, underground spaces, indoor and outdoor industrial spaces, and construction sites.

Outcome assessment

The primary outcome was the one-month survival rate with favorable neurological status (Cerebral Performance Category 1 or 2) [15]. Secondary outcomes included rates of bystander CPR and public access defibrillation (PAD) and one-month survival (Cerebral Performance Category 1 to 5).

Statistical analysis

We assessed the overall incidence of OHCA in Japan during the study period of 2016-2021. The annual incidence rate per 100,000 persons was calculated using Japanese estimated population data for each year, and its trend was calculated using a Poisson regression model. Categorical variables are presented as numbers with proportions, and χ^2^ tests were used to assess group differences. Continuous variables are summarized as medians with interquartile ranges, and the Wilcoxon-Mann-Whitney U test was used to evaluate differences between groups. All statistical analyses were performed using JMP Pro version 17 (SAS Institute, Cary, NC, USA). All tests were two-tailed, and the significance level was set at P<0.05.

The number of OHCA cases in the workplace during the study period determined the sample size. Workplace OHCAs were grouped by pre-pandemic (10,908 cases) and pandemic period (5,456 cases). The outcomes of the two groups were compared. Subgroup analyses were performed similarly for office-onset versus non-office-onset cases and for cases occurring in COVID-19 infection high-burden regions versus low-burden regions.

We conducted a two-variate logistic regression analysis using the month and two-year periods as variables to examine the effect of the pandemic on the outcomes and used χ^2^ tests to verify the interaction. Multivariable logistic regression analysis was used to assess the outcome factors related to the pandemic; the odds ratio (OR) and its 95% confidence interval (CI) were calculated. The potential confounding factors of workplace OHCA based on previous studies were unclear. Therefore, we created a model that included all independent factors of characteristics using this study related to the survival of OHCA and the outcomes themselves and applied a backward (variable reduction) regression procedure. To avoid excluding potential candidate variables, the model with the lowest AIC (Akaike Information Criterion) was selected as the final model. In this case, the four outcome factors (one-month survival with favorable neurological status, bystander CPR, PAD, and one-month survival) were fixed variables. The final model included 13 independent factors, which seemed to be a reasonable number considering the sample size. The factors selected in the final model were included in the two subgroup analyses. The fitness of the models was assessed by the area under the curve (AUC) in the receiver operating characteristic curve (ROC).

## Results

Study population

**Figure 2:**
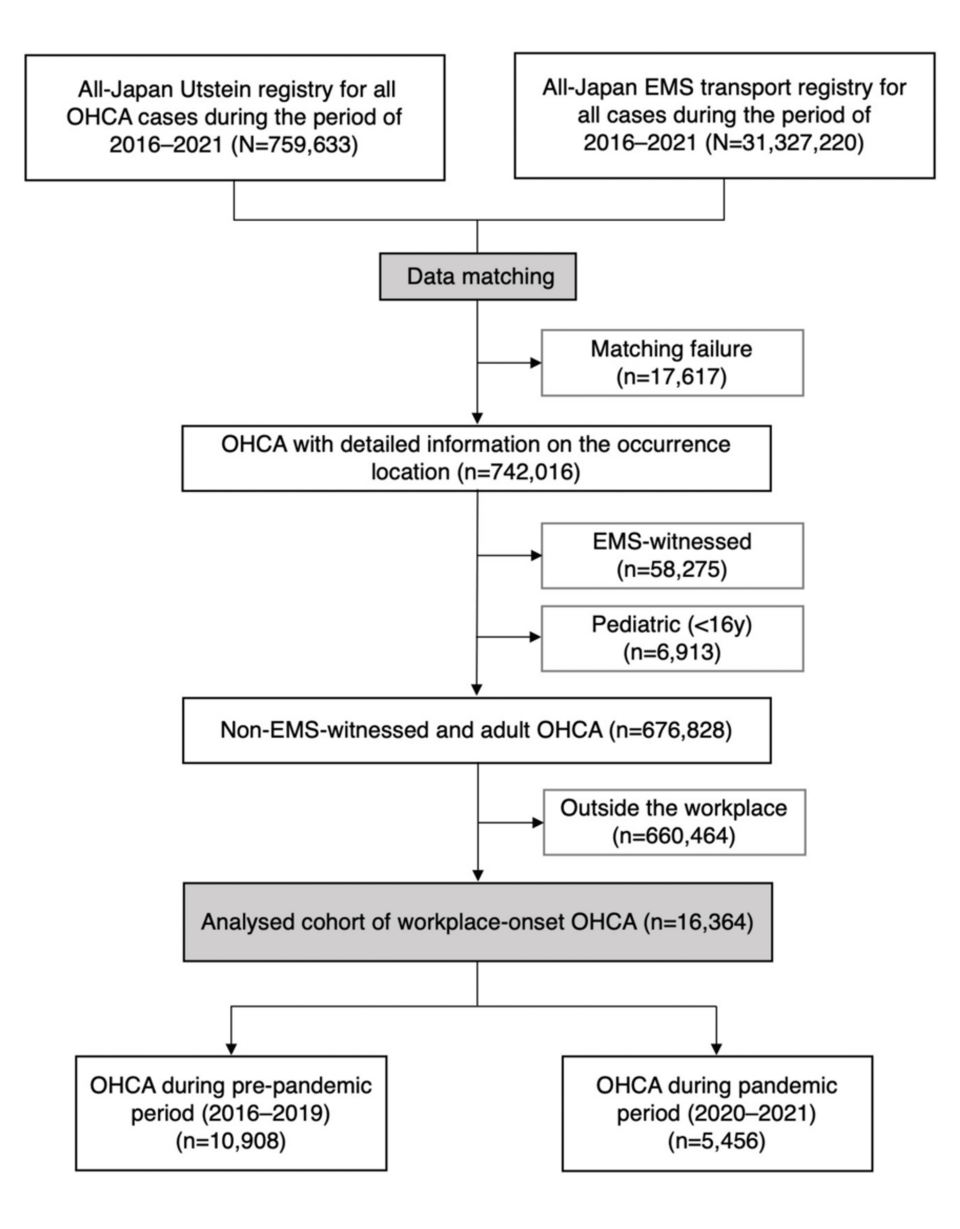
Flow diagram of the patient selection OHCA: out-of-hospital cardiac arrest; EMS: emergency medical service

A total of 759,633 OHCA cases from the Utstein Registry were matched with the EMS Transport Registry of 31,327,220 cases, and 17,617 data points were excluded due to failed matches. Next, we excluded 58,275 EMS-witnessed cases, 6,913 pediatric cases (<16 years), and 660,464 non-workplace cases. The final cohort consisted of 16,364 cases. Of these, 10,908 cases were in the pre-pandemic period (2016-2019), and 5,456 were in the pandemic period (2020-2021) (Figure 2). The breakdown of workplaces was 16.2% (2,642) in offices, 24.2% (3,953) at indoor construction sites, 16.0% (2,614) at outdoor construction sites, 11.9% (1,953) at factories, 21.0% (3,430) in agricultural areas, and 10.8% (1,772) in others.

There was no change in the annual incidence of all OHCAs during the study period (Table 1).

**Table 1:** Yearly trend in the incidence of all OHCA in Japan OHCA: out-of-hospital cardiac arrest Values represent incidence per 100,000 persons per year. We assessed the overall incidence of OHCA in Japan during the study period, 2016-2021: the annual incidence rate per 100,000 persons was calculated using Japanese estimated population data for each year, and its trend was calculated using a Poisson regression model.

	Year						P for trend
	2016	2017	2018	2019	2020	2021	
All	97.4	100.2	100.9	99.9	99.8	102.5	P=0.99
Male	114.2	117.6	118.8	117.7	118.7	121.9	P=0.99
Female	81.4	83.8	84.0	83.2	82.0	84.2	P=0.99

**Figure 3:**
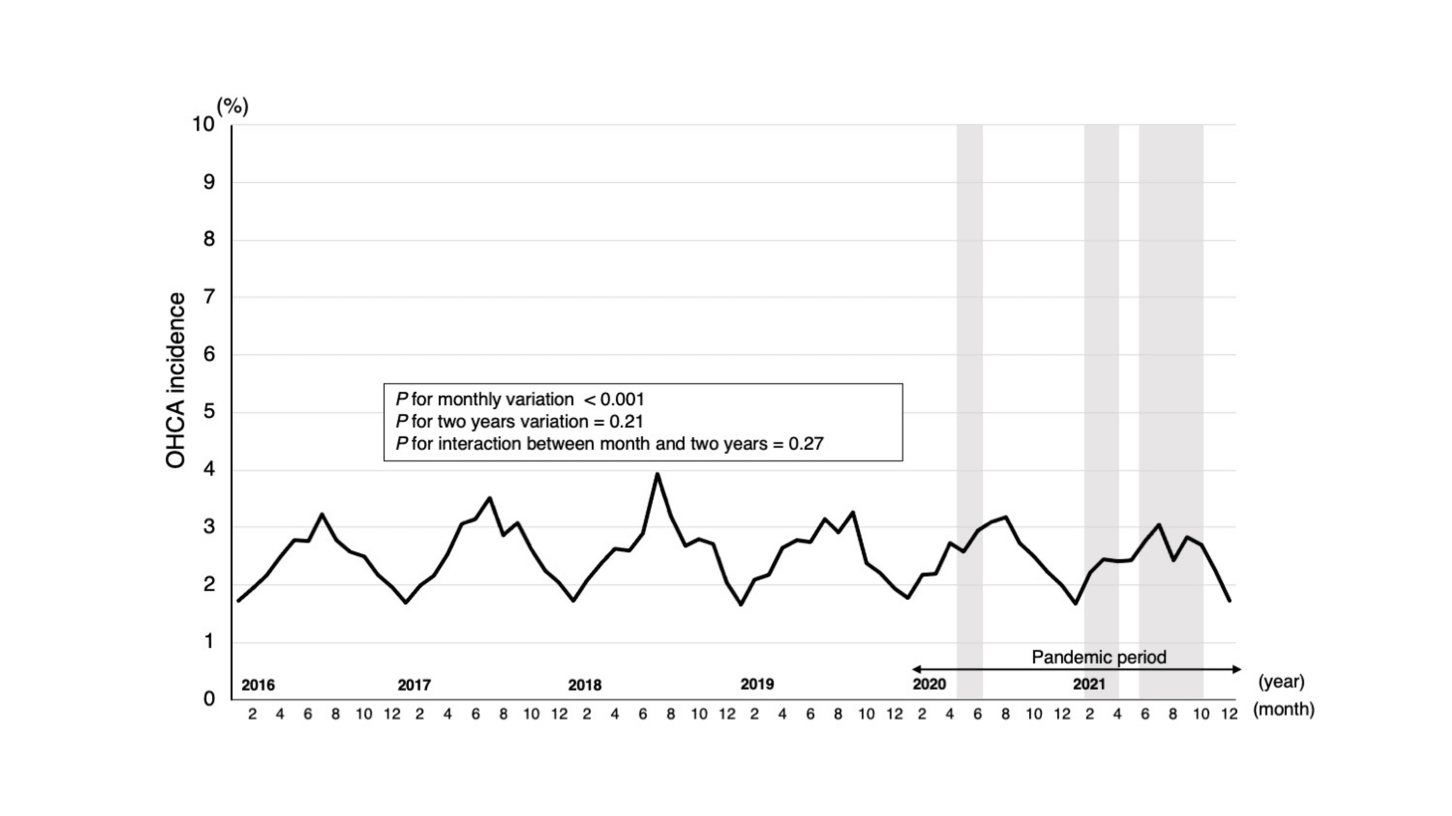
Trend of OHCAs occurring in the workplace before and during the COVID-19 pandemic Trends in the monthly incidence of workplace OHCA at work were analyzed for the four-year pre-pandemic and two-year pandemic periods. The monthly trends over the six-year study period and the two-year basis trends were evaluated using the χ^2^ test. A two-variable logistic regression analysis was conducted, including the month and two years. χ^2^ test was conducted to verify these relationships and confirm the trend. The grey area represents the three emergency declaration periods. First (47/47 prefectures): April 7, 2020-May 25, 2020; second (11/47 prefectures): January 8, 2021-March 21, 2021; third (21/47 prefectures): April 25, 2021-September 30, 2021. OHCA: out-of-hospital cardiac arrest

The annual incidence of non-EMS-witnessed adult OHCAs occurring in the workplace also remained in the 2% range. The trend of OHCA incidence showed significant monthly variations (P for monthly variation<0.001) (Figure 3). However, a two-year basis (biennial) analysis showed no significant change (P for two years variation=0.21). Furthermore, the monthly changes were not altered biennially (P for interaction=0.27).

Effect of COVID-19 pandemic on workplace OHCA characteristics and outcomes

The characteristics of workplace OHCA cases were compared between the pandemic and pre-pandemic periods. The proportion of patients older than 70 years (crude odds rate (cOR), 95% CI: 1.16, 1.08-1.25; P<0.001) and presumed cardiac etiology (cOR, 95% CI: 1.07, 1.00-1.15; P=0.04) were slightly higher during the pandemic. While suicide rates as a cause of cardiac arrest decreased, medical cardiac arrests increased. There was no significant change in bystander witnessing rate. The rates of dispatchers-assisted CPR (DA-CPR) (pandemic vs. pre-pandemic period: 56.2% vs. 54.5%; cOR, 95% CI: 1.07, 1.01-1.14; P=0.04) increased significantly during the pandemic. During the pandemic period, the rate of advanced airway management by EMS decreased (cOR, 95% CI: 0.84, 0.78-0.89; P<0.001), but the rate of adrenaline administration increased (cOR, 95% CI: 1.36, 1.26-1.45; P<0.001). Both EMS response interval and EMS transportation interval were prolonged during the pandemic period. The rates of bystander CPR (pandemic vs. pre-pandemic period: 58.1% vs. 55.9%; cOR, 95% CI: 1.10, 1.02-1.16; P<0.001) increased significantly during the pandemic. Notably, the rate of compression-only CPR increased, whereas that of conventional bystander CPR decreased. The rate of PAD provision showed no predominant association between the two periods (8.2% vs. 7.9%; cOR, 95% CI: 1.04, 0.92-1.18; P=0.50). Regarding survivals, neither the one-month survival rate (14.7% vs. 15.3%; cOR, 95% CI: 0.96, 0.88-1.05; P=0.38) nor the one-month survival rate with favorable neurological function (10.4% vs. 11.0%; cOR, 95% CI: 0.95, 0.85-1.05; P=0.31) showed a clear association with the pandemic period (Table 2).

**Table 2:** Characteristics and outcomes of OHCA occurring in the workplace OHCA: out-of-hospital cardiac arrest; IQR: interquartile range; PAD: public access defibrillation; CPR: cardiopulmonary resuscitation; DA-CPR: dispatchers-assisted CPR; EMS: emergency medical service; NA: not applicable Categorical variables are presented as (numbers) with proportions, and χ^2^ tests were used to assess group differences. Continuous variables are summarized as medians with interquartile ranges, and the Mann-Whitney U test was used to evaluate differences between groups. Using a univariate logistic regression analysis, crude odds ratio (OR) and 95% confidence interval (CI) were calculated with pre-pandemic periods as the reference. ^a^ Water, traffic, labor, fire accidents, and general injury. ^b^ Providing supraglottic airway devices or endotracheal intubation. ^c^ Time interval between emergency call receipt and EMS contact to patient. ^d^ Time interval between vehicle accommodation and EMS arrival at hospital. ^e^ Tertiary emergency medical institutions that provide advanced medical care for critically ill patients. In 2022, there were 299 facilities nationwide.

Variable	Pre-pandemic period (2016-2019) n=10,908	Pandemic period (2020-2021) n=5,456	P-value	Crude OR (95% CI)
Characteristics				
Regions with a high-burden of infection	21.4 (2,339)	20.3 (1,107)	0.09	0.93 (0.86-1.01)
Daytime (6:00 a.m. to 7:59 p.m.)	90.9 (9,915)	91.2 (4,976)	0.52	1.04 (0.93-1.08)
Office	15.7 (1,710)	17.1 (922)	0.02	1.11 (1.02-1.21)
Male	87.4 (9,528)	87.5 (4,773)	0.82	1.01 (0.92-1.12)
Age (IQR)	62 (51-71)	62 (52-72)	0.02	NA
Older adult (≥70 y)	29.1 (3,175)	32.3 (1,762)	<0.001	1.16 (1.08-1.25)
Witness status	NA	NA	0.10	NA
Unwitnessed	55.9 (6,100)	56.9 (3,102)	NA	1.04 (0.97-1.11)
Friend or colleague-witnessed	33.3 (3,636)	33.4 (1,824)	NA	1.01 (0.94-1.08)
Other-witnessed	5.1 (551)	4.7 (257)	NA	0.89 (0.80-0.99)
Presumed cardiac etiology	61.0 (6,651)	62.6 (3,416)	0.04	1.07 (1.00-1.15)
Cause classification	NA	NA	0.13	NA
Medical	70.1 (7,649)	71.6 (3,907)	NA	1.07 (1.00-1.15)
Accidental^a^	21.6 (2,358)	27.7 (1,511)	NA	0.96 (0.89-1.04)
Suicidal or self-harm	7.3 (800)	6.5 (353)	NA	0.87 (0.77-0.99)
Other non-medical	0.9 (101)	0.9 (50)	NA	0.99 (0.70-1.39)
Shockable initial rhythm	26.9 (2,930)	26.5 (1,445)	0.61	0.98 (0.91-1.06)
DA-CPR attempt	54.5 (5,943)	56.2 (3,064)	0.04	1.07 (1.01-1.14)
Advanced airway management^b^ by EMS	51.0 (5,558)	46.5 (2,537)	<0.001	0.84 (0.78-0.89)
Adrenaline administration by EMS	28.4 (3,098)	35.0 (1,907)	<0.001	1.36 (1.26-1.45)
EMS response time interval^c^, min (IQR)	10 (8-13)	11 (8-14)	<0.001	NA
Transportation time interval^d^, min (IQR)	23 (17-30)	24 (18-31)	<0.001	NA
Transportation to emergency and critical care center^e^	53.0 (5,785)	55.1 (3,006)	0.01	1.09 (1.02-1.16)
Outcomes				
PAD provision	7.9 (857)	8.2 (445)	0.50	1.04 (0.92-1.18)
Bystander CPR	NA	NA	<0.001	NA
No bystander CPR	44.1 (4,811)	41.9 (2,286)	NA	0.91 (0.86-0.98)
Compression-only	49.0 (5,348)	53.3 (2,910)	NA	1.19 (1.11-1.27)
Conventional	6.9 (749)	4.8 (260)	NA	0.68 (0.59-0.78)
One-month survival	15.3 (1,664)	14.7 (803)	0.38	0.96 (0.88-1.05)
One-month survival with favorable neurological status	11.0 (1,195)	10.4 (569)	0.31	0.95 (0.85-1.05)

Multivariable analysis also revealed an association between an increase in bystander CPR, and in particular, an increase in compression-only CPR, and the pandemic (adjusted OR, 95% CI: 1.55, 1.33-1.81; P<0.001 conventional as reference) (Table 3).

**Table 3:** Factors associated with the COVID-19 pandemic PAD: public access defibrillation; CPR: cardiopulmonary resuscitation; EMS: emergency medical service; IQR: interquartile range; OR: odds ratio; CI: confidence interval; NA: not applicable The logistic regression model was created by a variable reduction method based on the Akaike criterion. The initial model for the reduction method included all variables in Table 1. PAD provision, bystander CPR, one-month survival, and one-month survival with favorable neurological status were treated as fixed variables. Factors selected in the final model were regions with a high-burden region of infection, office setting, witness status, presumed cardiac etiology, shockable initial rhythm, advanced airway management by EMS, adrenaline administration by EMS, transportation time interval, PAD provision, bystander CPR, one-month survival, and one-month survival with favorable neurological status. The area under the curve for this final model is 0.57. ^a^ Providing supraglottic airway devices or endotracheal intubation. ^b^ Time interval between vehicle accommodation and EMS arrival at hospital.

Valuable	P-value	Adjusted OR (95% CI)
Characteristics		
Regions with a high-burden of infection	0.07	1.07 (0.99-1.17)
Office	<0.001	1.18 (1.07-1.29)
Older adult (≥70 y)	<0.001	1.18 (1.10-1.28)
Witness status	0.01	NA
Unwitnessed	NA	1.02 (0.94-1.10)
Friend or colleague-witnessed	NA	NA
Other-witnessed	NA	0.86 (0.76-0.97)
Presumed cardiac etiology	0.04	1.08 (1.00-1.17)
Shockable initial rhythm	0.13	0.93 (0.84-1.02)
Advanced airway management^a^ by EMS	<0.001	0.76 (0.71-0.81)
Adrenaline administration by EMS	<0.001	1.45 (1.34-1.56)
Transportation time interval^b^, min (IQR)	0.001	1.00 (1.00-1.01)
Outcomes		
PAD provision	0.09	1.13 (0.98-1.31)
Bystander CPR	<0.001	NA
No bystander CPR	NA	0.88 (0.82-0.94)
Compression-only	NA	NA
Conventional	NA	0.64 (0.55-0.75)
One-month survival	0.98	1.00 (0.85-1.19)
One-month survival with favorable neurological status	0.60	0.95 (0.79-1.15)

**Figure 4:**
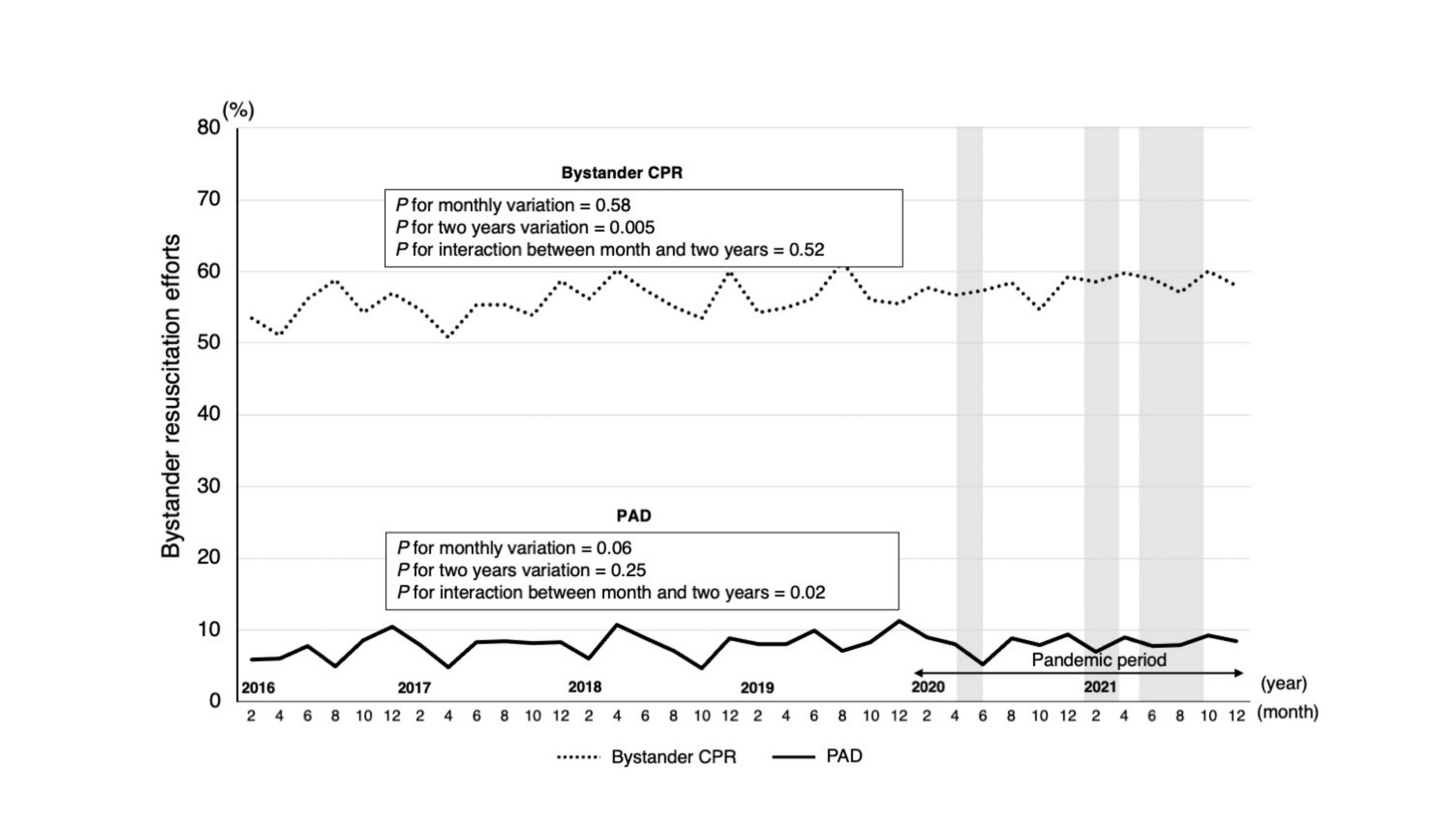
Trend of bystander resuscitation efforts in the workplace before and during the COVID-19 pandemic Trends in bystander CPR and PAD provision rates were analyzed for the four-year pre-pandemic and two-year pandemic periods. The monthly trends over the six-year study period and the two-year basis trends were evaluated using the χ^2^ test. A two-variable logistic regression analysis was conducted, including the month and two years. χ^2^ test was conducted to verify these relationships and confirm the trend. The grey area represents the three emergency declaration periods. First (47/47 prefectures): April 7, 2020-May 25, 2020; second (11/47 prefectures): January 8, 2021-March 21, 2021; third (21/47 prefectures): April 25, 2021-September 30, 2021. CPR: cardiopulmonary resuscitation; PAD: public access defibrillation

Additionally, there was no association between the pandemic and the rate of PAD provision (adjusted OR, 95% CI: 1.13, 0.98-1.31; P=0.09), one-month survival rate (adjusted OR, 95% CI: 1.00, 0.85-1.19; P=0.98), or neurological one-month survival rate (adjusted OR, 95% CI: 0.95, 0.79-1.15; P=0.60). The trend analysis of the six-year study period divided into two-year units revealed that only bystander CPR changed significantly (P for two years variation=0.005) (Figure 4).

**Figure 5:**
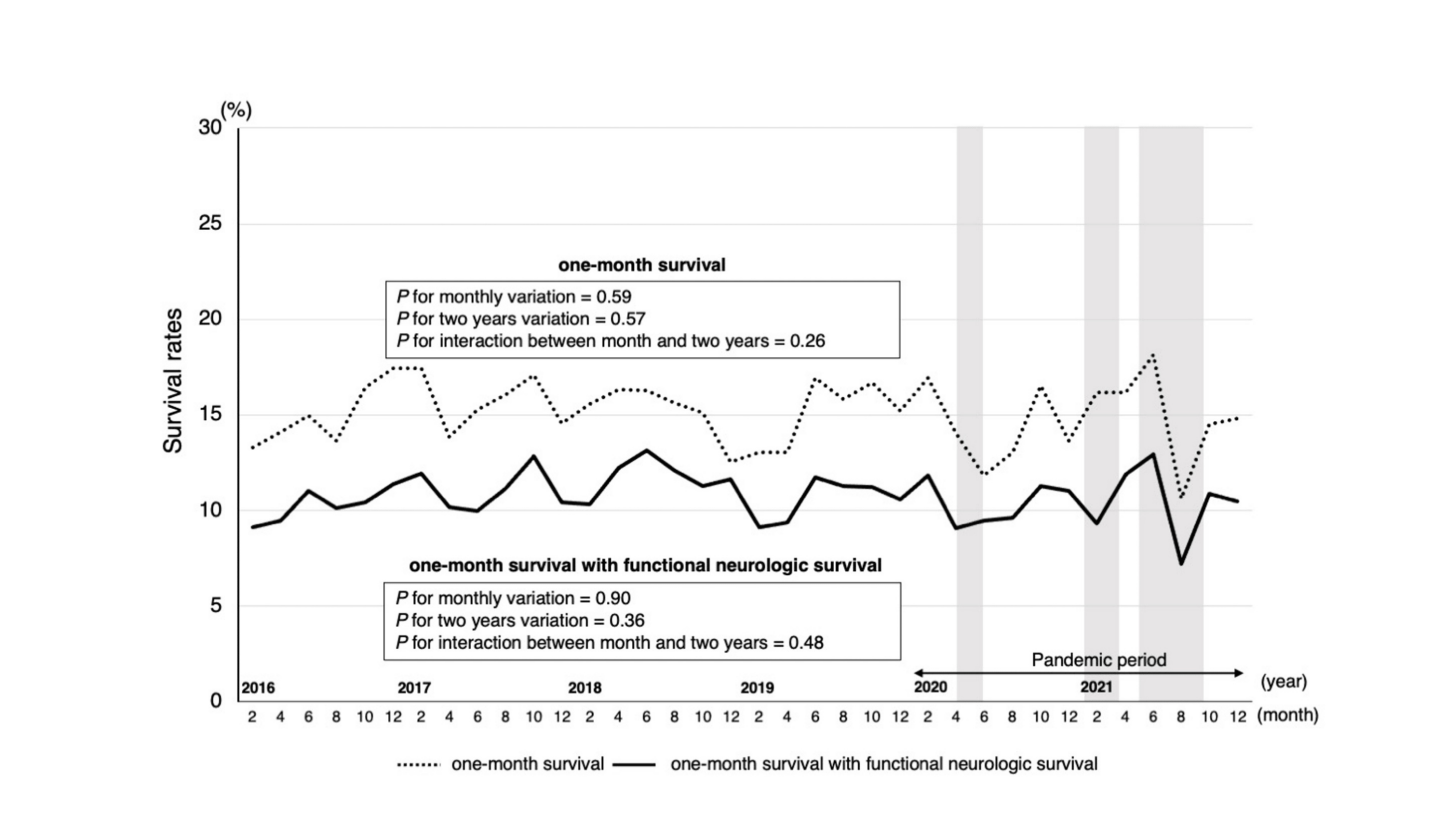
Trend of survival rates in the workplace before and during the COVID-19 pandemic Trends in one-month survival and one-month favorable neurological survival rates were analyzed for the four-year pre-pandemic and two-year pandemic periods. The monthly trends over the six-year study period and the two-year basis trends were evaluated using the χ^2^ test. A two-variable logistic regression analysis including the month and two years was conducted. The χ^2^ test was performed to verify these relationships and confirm the trend. The grey area represents the three emergency declaration periods. First (47/47 prefectures): April 7, 2020-May 25, 2020; second (11/47 prefectures): January 8, 2021-March 21, 2021; third (21/47 prefectures): April 25, 2021-September 30, 2021.

No significant change in the rate of PAD (P for two years variation=0.25), one-month survival rate (P for two years variation=0.57), or favorable neurological survival (P for two years variation=0.36) was observed (Figure 5). Furthermore, the monthly changes in only PAD were altered biennially (P for interaction=0.02), but there were no monthly changes in bystander CPR, PAD, one-month survival, or favorable neurological survival.

Characteristics and outcomes of OHCAs occurring in offices and high COVID-19 burden regions

As a subgroup analysis, examination of the pandemic’s impact on office-onset OHCAs revealed increased occurrences among older adults in both groups (offices and non-offices) (Table 4). During the pandemic, bystander CPR, especially compression-only CPR, increased only outside offices (cOR, 95% CI: 1.19, 1.11-1.28; P<0.001), with no change in the incidence of PAD rates and survival rates in either group (offices and non-offices). However, no significant association was found between all the factors of the characteristics and whether or not it was an office setting.

**Table 4:** Differences in the impact of the pandemic on OHCA occurring in the office and non-office OHCA: out-of-hospital cardiac arrest; PAD: public access defibrillation; CPR: cardiopulmonary resuscitation; EMS: emergency medical service; IQR: interquartile range; NA: not applicable Categorical variables are presented as numbers with proportions, and χ^2^ tests were used to assess group differences. Continuous variables are summarized as medians with interquartile ranges, and the Mann-Whitney U test was used to evaluate differences between groups. Using a univariate logistic regression analysis, crude odds ratio (OR) and 95% confidence interval (CI) were calculated with pre-pandemic periods as the reference. ^a^ Two-variate interaction test using logistic regression analysis, including pandemic period and working area classification. ^b^ Providing supraglottic airway devices or endotracheal intubation. ^c^ Time interval between vehicle accommodation and EMS arrival at hospital.

Variable	Working areas								P for interaction^a^
	Office				Non-office				
	Pre-pandemic period (2016-2019) N=1,710	Pandemic period (2020-2021) N=932	P-value	Crude OR (95% CI)	Pre-pandemic period (2016-2019) N=9,198	Pandemic period (2020-2021) N=4,524	P-value	Crude OR (95% CI)	
Characteristics									
Regions with a high-burden of infection	27.4 (469)	25.3 (236)	0.25	0.90 (0.75-1.08)	20.3 (1870)	19.3 (871)	0.14	0.93 (0.85-1.02)	0.69
Older adult (≥70 y)	15.6 (267)	18.7 (174)	0.04	1.24 (1.01-1.53)	31.6 (2,908)	35.1 (1,588)	<0.001	1.17 (1.09-1.26)	0.61
Witness status	NA	NA	0.32	NA	NA	NA	0.03	NA	0.40
Unwitnessed	37.3 (638)	36.5 (368)	NA	0.91 (0.77-1.07)	59.4 (5,462)	60.4 (2,734)	NA	0.96 (0.89-1.03)	NA
Friend or colleague-witnessed	52.5 (898)	49.5 (461)	NA	0.89 (0.75-1.04)	29.7 (2,732)	30.1 (1,363)	NA	1.02 (0.94-1.10)	NA
Other-witnessed	10.2 (174)	11.1 (103)	NA	1.10 (0.85-1.42)	10.9 (1,004)	9.4 (427)	NA	0.85 (0.75-0.96)	NA
Presumed cardiac etiology	73.0 (1,248)	75.5 (704)	0.16	1.14 (0.95-1.37)	58.7 (5,403)	60.0 (2,712)	0.14	1.06 (0.98-1.14)	0.41
Shockable initial rhythm	38.9 (665)	38.8 (362)	1.00	1.00 (0.93-1.18)	24.6 (2,265)	23.9 (1,083)	0.39	0.96 (0.89-1.05)	0.71
Advanced airway management^b^ by EMS	54.6 (935)	43.9 (409)	0.46	0.94 (0.80-1.11)	52.0 (4782)	47.0 (2,128)	<0.001	0.82 (0.76-0.88)	0.12
Adrenaline administration by EMS	25.2 (430)	28.4 (265)	0.07	1.18 (0.99-1.42)	29.0 (2668)	36.3 (1,642)	<0.001	1.39 (1.29-1.50)	0.10
Transportation time interval^c^, min (IQR)	21 (16-26)	22 (17-28)	<0.001	NA	23 (17-31)	24 (18-32)	<0.001	NA	0.18
Outcomes									
PAD provision	14.3 (244)	13.3 (124)	0.50	0.92 (0.73-1.16)	6.7 (613)	7.1 (321)	0.35	1.07 (0.93-1.23)	0.28
Bystander CPR	NA	NA	0.15	NA	NA	NA	<0.001	NA	0.39
No bystander CPR	38.0 (650)	36.1 (336)	NA	0.92 (0.78-1.08)	45.2 (4,161)	43.1 (1,950)	NA	0.92 (0.85-0.99)	NA
Compression-only	53.2 (910)	56.8 (529)	NA	1.15 (0.98-1.36)	48.3 (4,438)	52.6 (2,381)	NA	1.19 (1.11-1.28)	NA
Conventional	8.8 (150)	7.3 (67)	NA	0.81 (0.60-1.09)	6.5 (599)	4.3 (193)	NA	0.63 (0.54-0.76)	NA
One-month survival	27.3 (467)	28.2 (263)	0.62	1.05 (0.88-1.25)	13.0 (1,197)	11.9 (540)	0.07	0.91 (0.81-1.01)	0.18
One-month survival with favorable neurological status	21.5 (368)	20.8 (194)	0.67	0.96 (0.79-1.17)	9.0 (827)	8.3 (375)	0.17	0.92 (0.81-1.04)	0.70

Furthermore, in infection high-burden regions, we observed an increase in the proportion of advanced airway management by EMS and an increase in EMS transportation intervals during the pandemic period (Table 5). We also found a decreasing trend in PAD provision rates during the pandemic only in high-burden regions (pandemic vs. pre-pandemic period: 8.4% vs. 10.0%; cOR, 95% CI: 0.83, 0.64-1.06; P=0.14), with an increasing trend in low-burden regions (8.1% vs. 7.3%; cOR, 95% CI: 1.12, 0.98-1.29; P=0.10) (interaction P=0.03). The rate of compressions-only CPR was significantly higher during the pandemic in both high-burden (cOR, 95% CI: 1.20, 1.04-1.38; P=0.007) and low-burden regions (cOR, 95% CI: 1.18, 1.10-1.27; P<0.001). Furthermore, a non-significant difference in one-month survival and neurologically favorable survival was observed in both groups during the pandemic.

**Table 5:** Differences in the impact of pandemics on workplace-onset OHCA between regions with a high burden of infection and other regions OHCA: out-of-hospital cardiac arrest; PAD: public access defibrillation; CPR: cardiopulmonary resuscitation; EMS: emergency medical service; IQR: interquartile range; NA: not applicable Categorical variables are presented as numbers with proportions, and χ^2^ tests were used to assess group differences. Continuous variables are summarized as medians with interquartile ranges, and the Mann-Whitney U test was used to evaluate differences between groups. Using a univariate logistic regression analysis, crude odds ratio (OR) and 95% confidence interval (CI) were calculated with pre-pandemic periods as the reference. ^a^ Prefectures where a state of emergency was declared. ^b^ Prefectures where a state of emergency was not declared. ^c^ Two-variate interaction test using logistic regression analysis, including pandemic period and classification of infection burden regions. ^d^ Providing supraglottic airway devices or endotracheal intubation. ^e^ Time interval between vehicle accommodation and EMS arrival at hospital.

Variable	Region								P for interaction^c^
	Regions^a^ with a high-burden of infection (matched by date and prefecture)				Regions^b^ with a low-burden of infection (matched by date and prefecture)				
	Pre-pandemic period (2016-2019) N=2,339	Pandemic period (2020-2021) N=1,107	P-value	Crude OR (95% CI)	Pre-pandemic period (2016-2019) N=8,569	Pandemic period (2020-2021) N=4,349	P-value	Crude OR (95% CI)	
Characteristics									
Office	20.1 (469)	21.3 (236)	0.39	1.09 (0.91-1.29)	14.5 (1,241)	16.0 (696)	0.02	1.13 (1.02-1.24)	0.69
Older adult (≥70 y)	27.1 (634)	30.6 (339)	0.04	1.19 (1.01-1.39)	29.7 (2,541)	32.7 (1,423)	<0.001	1.15 (1.07-1.25)	0.75
Witness status	NA	NA	0.02		NA	NA	0.53	NA	0.11
Unwitnessed	54.6 (1,278)	56.3 (623)	NA	1.07 (0.93-1.23)	56.3 (4,822)	57.0 (2,479)	NA	1.04 (0.90-1.04)	NA
Friend or colleague-witnessed	34.6 (809)	35.6 (398)	NA	0.94 (0.81-1.09)	32.9 (2,822)	32.8 (1,426)	NA	0.99 (0.92-1.07)	NA
Others-witnessed	10.8 (252)	17.8 (86)	NA	1.43 (1.11-1.85)	10.8 (926)	10.2 (444)	NA	0.94 (0.83-1.06)	NA
Presumed cardiac etiology	64.8 (1,515)	65.7 (727)	0.62	1.04 (0.90-1.21)	59.9 (5,136)	61.8 (2,689)	0.04	1.08 (1.00-1.17)	0.64
Shockable initial rhythm	29.8 (698)	28.9 (320)	0.60	0.96 (0.82-1.12)	26.1 (2,232)	25.9 (1,125)	0.83	0.99 (0.91-1.08)	0.69
Advanced airway management^d^ by EMS	47.0 (1,100)	54.1 (599)	<0.001	1.33 (1.15-1.54)	52.0 (4,458)	44.6 (1,938)	<0.001	0.74 (0.69-0.80)	<0.001
Adrenaline administration by EMS	28.1 (657)	36.4 (403)	<0.001	1.47 (1.26-1.71)	28.5 (2,441)	34.6 (1,540)	<0.001	1.33 (1.23-1.43)	0.26
Transportation time interval^e^, min (IQR)	22 (17-29)	24 (18-31)	<0.001	NA	23 (17-30)	24 (18-31)	<0.001	NA	0.04
Outcomes									
PAD provision	10.0 (234)	8.4 (93)	0.14	0.83 (0.64-1.06)	7.3 (623)	8.1 (352)	0.10	1.12 (0.98-1.29)	0.03
Bystander CPR	NA	NA	0.007	NA	NA	NA	<0.001	NA	0.98
No bystander CPR	45.2 (1,056)	42.9 (475)	NA	0.91 (0.79-1.05)	43.8 (3,755)	41.6% (1,811)	NA	0.91 (0.85-0.98)	NA
Compression-only	47.8 (1,118)	52.3 (579)	NA	1.20 (1.04-1.38)	49.4 (4,230)	53.6% (2,331)	NA	1.18 (1.10-1.27)	NA
Conventional	7.1 (165)	4.8 (53)	NA	0.66 (0.48-0.91)	6.8 (584)	4.8% (207)	NA	0.68 (0.56-0.80)	NA
One-month survival	17.3 (404)	15.6 (173)	0.24	0.89 (0.73-1.08)	14.7 (1,260)	14.5% (630)	0.75	0.98 (0.89-1.09)	0.36
One-month survival with favorable neurological status	11.7 (273)	10.8 (119)	0.46	0.91 (0.73-1.15)	10.8 (922)	10.4% (450)	0.49	0.96 (0.85-1.08)	0.71

## Discussion

This nationwide, population-based study in Japan investigated the impact of the COVID-19 pandemic on workplace OHCA. Contrary to our initial hypothesis, the pandemic did not significantly influence survival rates for workplace OHCAs. Regarding bystander efforts, a slight increase in bystander CPR rates was observed during the pandemic, and PDA rates did not change. No increase in bystander CPR rates was observed during the declared emergency period, but a downward trend in PDA rates was observed. Furthermore, no significant interaction was identified between workplace type (office vs. non-office) and the pandemic on OHCA outcomes or bystander resuscitation efforts. This study is the first to identify the impact of the COVID-19 pandemic on workplace cardiac arrest.

Notably, these findings reveal that the workplace is an exemplary setting in which bystander CPR and AED use are actively provided, even during a pandemic, resulting in strong maintenance of the chain of survival. It is encouraging that such proactive resuscitation efforts may have maintained the number of patients surviving. Based on this workplace observation, it is important to identify other settings unaffected by the COVID-19 pandemic and further investigate their common characteristics. The results will provide important clues for planning and implementing effective strategies to maintain OHCA survival in the event of new infectious disease pandemics or other crises, such as disasters, that humanity may experience in the future.

There was no change in the OHCA incidence by year of occurrence in Japan during the study period. Moreover, a two-year basis trend analysis did not reveal a significant change in the incidence of workplace OHCA, and the impact of the pandemic was not evident. A meta-analysis suggested that OHCA incidence increased in some countries during the pandemic, while there was no change in other countries [16]. Further, the analysis that considered countries such as the United States and Spain, which had relatively high numbers of infected individuals, showed a decrease in the incidence of workplace OHCA during the pandemic period, from 1.0% to 0.4% [16]. Japan had a comparatively low number of infected individuals and did not implement an urban lockdown policy. These differences in incidence might have been influenced by regional variations in infection burden and control practices.

Previous meta-analyses have revealed that the COVID-19 pandemic dramatically reduced the survival rate of patients with OHCA [3,9], with a report of approximately 50% reduction in hospital discharge survival [16]. Furthermore, reports from Japan have also confirmed an obvious decrease in survival rates during the pandemic [17]. Possible reasons for this may include bystanders’ reluctance to perform CPR for fear of spreading the virus via aerosols [18,19], limited availability of public automated external defibrillators (AEDs), and disorganization of the emergency medical system, which delays the initiation of EMS resuscitation and transport [12]. In Japan, the Industrial Safety and Health Act mandates health management in the workplace [20], thus, workers are highly aware of industrial safety and health issues. Although limited during the pandemic, the introduction of regular CPR training programs and deployment of AEDs in the workplace had steadily increased [21,22] before the pandemic. The maintenance effect of previous efforts to improve CPR for bystanders may have contributed to the increase in bystander CPR in the workplace during the pandemic. Furthermore, in Japan, (1) temperature checks before entering the workplace led to workers being more thorough in managing their physical condition, decreasing concern about viral transmission; (2) in this study, 82% of the witnesses in the workplace were colleagues or friends, who are known to actively intervene with resuscitative attempts [23]. These two factors may have contributed to the lack of a decrease in bystander CPR. This suggests that the health and safety infrastructure in Japanese workplaces, including the implementation of thorough CPR training, proactive installation of AEDs, and strict occupational health and safety environments, may have contributed to maintaining survival rates.

The increased rate of chest-compression-only CPR may be due to the increasing trend of DA-CPR by dispatchers [24,25]. Dispatchers actively taught chest-compressions-only CPR during the pandemic to avoid the risk of infection transmission during mouth-to-mouth ventilation. The International Liaison Committee on Resuscitation (ILCOR) recommends conventional CPR because chest-compressions-only CPR may reduce survival rates [26]. However, our results suggest that chest-compressions-only CPR is effective in certain situations. The rate of advanced airway management (SGA or TI) by EMS was low during the pandemic, and the adrenaline administration rate was high. Previous observational findings have shown a survival advantage for bag-valve-mask (BVM) ventilation compared to advanced airway management [27]. Additionally, the effectiveness of prehospital adrenaline administration in terms of neurological prognosis has not been demonstrated [28]. Our results may suggest the effectiveness of BVM ventilation and adrenaline administration during a pandemic, but further research is needed. The risk of viral infection creates a dilemma regarding how best to manage CPR, but the increase in the rate of bystander CPR and the proactive use of BVM ventilation and adrenaline by EMS are welcome phenomena that reflect positively on society as a whole.

An estimated 802,000 available AEDs were installed in Japan (in 2022), the highest installation rate in the world (6,410 units per million population) [29]. Although there is no legal obligation to install AEDs in the workplace, many companies have actively installed them from the perspective of safety obligations. In fact, according to a 2016 small-scale survey, 88.4% of large companies and 40.8% of small to medium companies had AEDs [30]. Additionally, workers are more familiar with the locations of AEDs in the workplace than in public places, which may have contributed to their active use during the pandemic. Our findings also revealed a downward trend in PAD rates only under a declared state of emergency, which may be due to more restrictive attendance and movement restrictions within the workplace, preventing the use of AEDs. AEDs in the workplace should be placed in more effective locations accessible to all people, and the number and locations of AEDs in each workplace should be reviewed [31]. It is also important to follow the example of workplaces by raising health and safety awareness among the general public and by conducting regular and proactive CPR training sessions. Furthermore, the number of AEDs deployed should be increased, especially in multi-dwelling houses, including condominiums and apartments. The synergistic effect of these attempts could further strengthen the chain of survival.

The COVID-19 pandemic has slightly increased the proportion of older adults with OHCA within workplaces. While the exact reasons remain unclear, a potential explanation could be the re-employment of retired individuals due to pandemic-related labor shortages. Further, a slight increase in OHCA of presumed cardiac etiology was observed during the COVID-19 pandemic. This aligns with the established knowledge that disasters and stress temporarily increase the incidence of cardiovascular events [32]. In addition, the pandemic-induced decline in hospital visits for cardiovascular complaints could have contributed to increased OHCA risk [33]. Maintaining an adequate prehospital and hospital healthcare system during a pandemic is an important policy consideration.

This study had some limitations inherent to its observational design. First, we cannot completely establish causal relationships between the pandemic and the observed outcomes. Second, the matching process of the two registries failed to include 2.3% (17,617/759,633) of case failures. In addition, EMS-witnessed cases and pediatric cases were excluded. The exclusion of these patients may have affected the outcomes. Third, information regarding patients’ SARS-CoV-2 infection status was unavailable. Finally, data completeness, validity, and ascertainment bias were potential limitations, as in all epidemiological studies. However, the study’s strengths included using uniform data collection methods based on Utstein-style for cardiac arrest reporting, a large sample size, and a population-based design, all minimizing potential biases.

## Conclusions

The COVID-19 pandemic had no impact on the survival of patients with OHCA in workplaces and only a slight positive impact on bystander CPR. Furthermore, analyses of office settings and high-burden regions did not impact survival. The workplace environment appears to have maintained a relatively robust chain of survival during the pandemic, possibly due to the high level of health awareness in the workplace, as well as the proactive pre-existing CPR training and the installation of AEDs before the pandemic. The importance of establishing appropriate preparations and measures to improve the survival rate of patients in cardiac arrest cannot be overstated.

## References

[1] Wiersinga WJ, Rhodes A, Cheng AC, Peacock SJ, Prescott HC (2020). Pathophysiology, transmission, diagnosis, and treatment of coronavirus disease 2019 (COVID-19): a review. JAMA.

[2] Ahn C (2022). Suggestions for the focus of OHCA meta-analysis in the COVID-19 era. Resuscitation.

[3] Lim ZJ, Ponnapa Reddy M, Afroz A, Billah B, Shekar K, Subramaniam A (2020). Incidence and outcome of out-of-hospital cardiac arrests in the COVID-19 era: a systematic review and meta-analysis. Resuscitation.

[4] Scquizzato T, Landoni G, Paoli A (2020). Effects of COVID-19 pandemic on out-of-hospital cardiac arrests: a systematic review. Resuscitation.

[5] Norii T, Igarashi Y (2023). An unbroken ring of the chain of survival. Resuscitation.

[6] Pamidimukkala A, Kermanshachi S (2021). Impact of Covid-19 on field and office workforce in construction industry.

[7] Myat A, Song KJ, Rea T (2018). Out-of-hospital cardiac arrest: current concepts. Lancet.

[8] Soar J, Böttiger BW, Carli P (2021). European Resuscitation Council Guidelines 2021: adult advanced life support. Resuscitation.

[9] Bielski K, Szarpak A, Jaguszewski MJ (2021). The influence of COVID-19 on out-hospital cardiac arrest survival outcomes: an updated systematic review and meta-analysis. J Clin Med.

[10] Descatha A, Dagrenat C, Cassan P, Jost D, Loeb T, Baer M (2015). Cardiac arrest in the workplace and its outcome: a systematic review and meta-analysis. Resuscitation.

[11] Murakami Y, Iwami T, Kitamura T, Nishiyama C, Nishiuchi T, Hayashi Y, Kawamura T (2014). Outcomes of out-of-hospital cardiac arrest by public location in the public-access defibrillation era. J Am Heart Assoc.

[12] Ushimoto T, Yao S, Nunokawa C, Murasaka K, Inaba H (2023). Association between the COVID-19 pandemic in 2020 and out-of-hospital cardiac arrest outcomes and bystander resuscitation efforts for working-age individuals in Japan: a nationwide observational and epidemiological analysis. Emerg Med J.

[13] (2025). Transition of new coronavirus COVID-19 deaths per population by country. https://web.sapmed.ac.jp/canmol/coronavirus/death_e.html.

[14] (2025). Fire and Disaster Management Agency of the Ministry of Internal Affairs and Communications of Japan. https://www.fdma.go.jp/en/post1.html.

[15] Jennett B, Bond M (1975). Assessment of outcome after severe brain damage. Lancet.

[16] Baldi E, Klersy C, Chan P (2024). The impact of COVID-19 pandemic on out-of-hospital cardiac arrest: an individual patient data meta-analysis. Resuscitation.

[17] Katasako A, Yoshikawa Y, Noguchi T (2023). Changes in neurological outcomes of out-of-hospital cardiac arrest during the COVID-19 pandemic in Japan: a population-based nationwide observational study. Lancet Reg Health West Pac.

[18] Perkins GD, Morley PT, Nolan JP (2020). International Liaison Committee on Resuscitation: COVID-19 consensus on science, treatment recommendations and task force insights. Resuscitation.

[19] Jost D, Derkenne C, Kedzierewicz R (2020). The need to adapt the rescue chain for out-of-hospital cardiac arrest during the COVID-19 pandemic: experience from the Paris Fire Brigade Basic Life Support and Advanced Life Support teams. Resuscitation.

[20] (2025). Ministry of Health, Labor and Welfare of Japan. https://www.japaneselawtranslation.go.jp/en/laws/view/3440.

[21] Kobayashi D, Sado J, Kiyohara K (2020). Public location and survival from out-of-hospital cardiac arrest in the public-access defibrillation era in Japan. J Cardiol.

[22] Yamagishi Y, Oginosawa Y, Fujino Y (2021). Incidence of out-of-hospital cardiac arrests and survival rates after 1 month among the Japanese working population: a cohort study. BMJ Open.

[23] Tanaka Y, Maeda T, Kamikura T (2015). Potential association of bystander-patient relationship with bystander response and patient survival in daytime out-of-hospital cardiac arrest. Resuscitation.

[24] Tanaka Y, Taniguchi J, Wato Y, Yoshida Y, Inaba H (2012). The continuous quality improvement project for telephone-assisted instruction of cardiopulmonary resuscitation increased the incidence of bystander CPR and improved the outcomes of out-of-hospital cardiac arrests. Resuscitation.

[25] Goto Y, Funada A, Maeda T, Goto Y (2022). Association of dispatcher-assisted cardiopulmonary resuscitation with initial shockable rhythm and survival after out-of-hospital cardiac arrest. Eur J Emerg Med.

[26] Olasveengen TM, Semeraro F, Ristagno G (2021). European Resuscitation Council Guidelines 2021: basic life support. Resuscitation.

[27] Hasegawa K, Hiraide A, Chang Y, Brown DF (2013). Association of prehospital advanced airway management with neurologic outcome and survival in patients with out-of-hospital cardiac arrest. JAMA.

[28] Perkins GD, Ji C, Deakin CD (2018). A randomized trial of epinephrine in out-of-hospital cardiac arrest. N Engl J Med.

[29] (2025). Nationwide survey of AED sales and installation numbers [Website in Japanese]. http://grants.niph.go.jp/system/files/report_pdf/202009042A-buntan.pdf.

[30] (2025). Which has a higher rate of AED installation: large companies or small and medium-sized companies? [Website in Japanese]. https://gooday.nikkei.co.jp/atcl/report/14/091100031/090700418/.

[31] Kitamura T, Kiyohara K, Sakai T (2016). Public-access defibrillation and out-of-hospital cardiac arrest in Japan. N Engl J Med.

[32] Leor J, Poole WK, Kloner RA (1996). Sudden cardiac death triggered by an earthquake. N Engl J Med.

[33] Choudhary R, Gautam D, Mathur R, Choudhary D (2020). Management of cardiovascular emergencies during the COVID-19 pandemic. Emerg Med J.

